# A method to quantify infection and colonization of holm oak (*Quercus ilex*) roots by *Phytophthora cinnamomi*

**DOI:** 10.1186/1746-4811-8-39

**Published:** 2012-09-13

**Authors:** Francisco J Ruiz-Gómez, Rafael Sánchez-Cuesta, Rafael M Navarro-Cerrillo, Alejandro Pérez-de-Luque

**Affiliations:** 1 Evaluación y Restauración de Sistemas Agrícolas y Forestales” (RNM360). Departamento de Ingeniería Forestal. E.T.S. Ingeniería Agronómica y de Montes, Universidad de Córdoba, Córdoba, Spain; 2 Instituto de Investigación y Formación Agraria y Pesquera de Andalucía-IFAPA, Centro “Alameda del Obispo”, Área de Mejora y Biotecnología, Avda. Menédez Pidal, s/n, PO Box 3092, Córdoba, 14004, Spain

**Keywords:** Phytophthora, Holm oak decline, Microscopy, Histology, Root rot, Infection degree

## Abstract

*Phytophthora cinnamomi* Rands. is an important root rot pathogen widely distributed in the north hemisphere, with a large host range. Among others diseases, it is known to be a principal factor in the decline of holm oak and cork oak, the most important tree species in the “dehesa” ecosystem of south-western Spain. Previously, the focus of studies on *P. cinnamomi* and holm oak have been on molecular tools for identification, functional responses of the host, together with other physiological and morphological host variables. However, a microscopic index to describe the degree of infection and colonization in the plant tissues has not yet been developed. A colonization or infection index would be a useful tool for studies that examine differences between individuals subjected to different treatments or to individuals belonging to different breeding accessions, together with their specific responses to the pathogen. This work presents a methodology based on the capture and digital treatment of microscopic images, using simple and accessible software, together with a range of variables that quantify the infection and colonization process.

## Background

*Phytophthora cinnamomi* Rands. is an oomycete belonging to the family *Pythiaceae*[1] that causes root rot in many woody, herbaceous, and shrub species. This pathogen is one of the most common phytopathogens in nature, with the number of potential hosts estimated at more than 3000 [2]. *Phytophthora cinnamomi* plays a role as one of the main factors participating in the decline syndrome of *Quercus*[3-9].

Holm oak (*Quercus ilex* L.) is the most common forest species in the Mediterranean basin and its surrounds [10], and is the most widespread species in Spain covering an area of over 4 million hectares [11]. This oak is the main species of the “dehesa” forest systems in the south-western portion of the Iberian Peninsula, which is of vital importance to animal husbandry in these ecosystems. Moreover, holm oak is one of the *Quercus* species with the highest susceptibility to *P. cinnamomi*[12-14], which accounts for the importance of studying the interaction between these species, especially considering the growing number of cases of declining holm oaks that have been detected in the “dehesas” since the 1990s.

Previous studies have used different approaches to explore pathogen-host relationships, that could be classified as (i) basic determination techniques, (ii) molecular techniques, (iii) morphophysiologic variable measurements, and (iv) experiments based on survival under stressful conditions. Basic techniques include the microscopic observation of isolated pathogens from infected tissue [4,6,9,15]. Currently, pathogen detection trough molecular analysis is an important field of study. ELISA, PCR or isozyme patterns have recently been used in this approach [2]. Morphophysiological studies include experiments on sapling development under controlled conditions [7,16], and have assessed the effectiveness of potential treatments, such as phosphonates in the presence or absence of the pathogen [8,11,17]. Moreover, it is relevant to highlight practical-focused experiments, which are based on the management and modification of novel and traditional cultural practices [18-20].

Several studies have been conducted to assess the pathogen-host interaction at a histological level [21-24], but no quantitative index that assesses the extent of *P. cinnamomi* infection and colonization at the tissue level has been presented for any forest species, as the morphological characteristics of oomycetes and their way of life render it difficult to apply this type of index [1,2].

In the study of fungal phytopathogens, it is common to use indices such as the number of spores, survival structures, stem or trunk lesions, pustules, and other reproductive structures that are present in the affected tissues [25-27]. These indices assess the level of infection in tissues and can reveal differences between individuals subjected to specific treatments or that exhibit different levels of resistance or susceptibility to the pathogen. An alternative method for quantifying the level of tissue infection and colonisation by pathogens includes a combination of microscopic techniques and digital analysis of images, and includes the application of stereological techniques in microscopy [28,29]. The use of stereological techniques in histological analysis requires the use of many equations and specialized statistical processing, which entails a highly specialized staff and a substantial investment of time and resources [29]. However, certain plant tissues present a level of structural simplicity that makes the precise digitalization of their structures easy through the use of simple computer tools. This allows for the transformation of the structures that result from the interactions between the host and the pathogen into quantitative data.

The objective of this study was to establish a specific methodology to determine the level of *P. cinnamomi* infection and colonisation in *Q. ilex* root tissues by applying computer techniques to process microscopic images, allowing us to obtain quantitative data and indices.

## Results

### Oomycete localization

**Figure 1:**
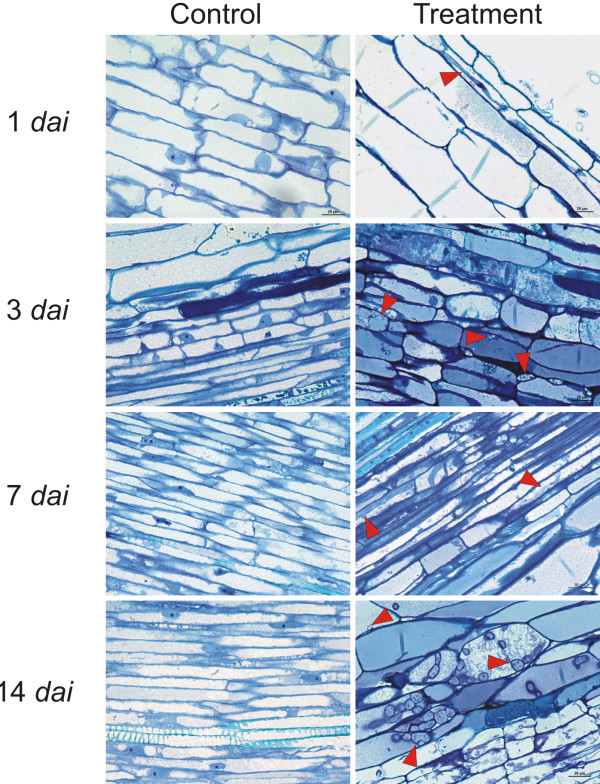
**Comparison of images of treated and control root sections over time (1–14 *dai*).** All the sections represent vascular tissue, except the images from 1 *dai*, which belong to the outer cortex (at this sampling time, the pathogen was not found in other sections). Some pathogen structures are indicated by red arrowheads in all the treated sections

**Figure 2:**
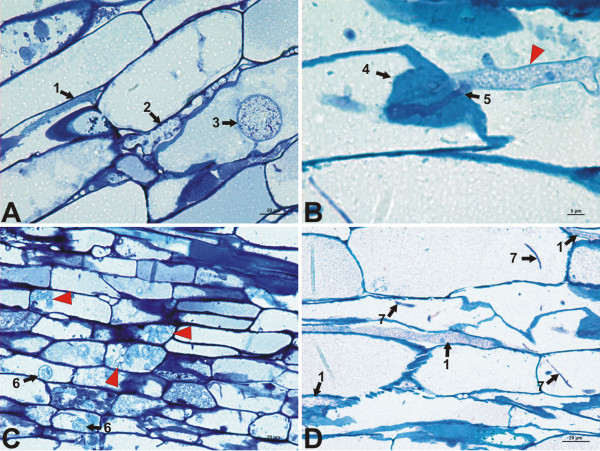
**Pathogen structures identified in longitudinal root sections. A**, Root cortical tissue. Several structures can be identified, such as primary hyphae (1), botryose hyphae (2), and survival structures such as chlamydospores (3). **B**, Hyphae observed at high magnification (x1000) (arrowhead). The pathogen penetrates the host cell through the cellulose wall. Accumulation of well stained cell wall material (4) (possibly papillae) is observed surrounding the penetration hyphae (5). **C**, Invasion of the central cylinder parechymatic tissue of the root by the pathogen 14 days after inoculation. Botryose hyphae that grow haphazardly were found inside the cells (arrowheads). At this time, we found immature or small chlamydospores in the parenchymatous cells (6). **D**, Several hyphae (1) and structures resembling finger-like haustoria (7) in cortical cells.

The staining method resulted in clearly differentiated pathogen structures, which stained to blue-violet, while most of the plant structures dyed dark blue. *Phytophthora cinnamomi* was observed in all of the sections for each sampling period, while it was not present in the control plant roots (Figure 1). We found aseptate hyphae (Additional files 1 and 2) with diameters ranging from 6 to 9 μm. From 3 *dai* (days after inoculation), it was common to find well developed hyphae in the cortex, in which cytosolic material was clearly distinguished and where we could observe nuclear material (Figure 2A, B, D). Where the pathogen could be found, we observed a type of hyphal structure inside the cells with a diameter ranging from 2 to 3 μm, that could resemble filiform haustoria, or fibrous finger-shaped haustoria. These structures, despite their small size, were easily distinguishable because of their uniform coloration, more purple than blue (Figure 2D). We also localized chlamydospores, which were frequent in the cortex of 14-*dai* samples (Figure 2A). At this time, a large number of specific structures of *P. cinnamomi* were localized inside the parenchyma cells of the vascular cylinder. These structures might be identified as botryose hyphal swellings or incipient chlamydospores (Figure 2C). We did not find structures for sexual reproduction (gametangia) or sexual spores (oospores).

In the sample sections from 1 *dai*, the oomycete was found almost entirely in the most external areas of the cortex and in the epidermis of the root, whereas only a few scattered structures in the parenchyma cells of the stele were found. Structures within the fibrotracheids of the xylem of the stele were not observed. However, at 3 *dai*, the oomycete was already present throughout the section, with a considerable increase in the structures in the cortex, although still rare within the fibrotracheids.

From 7 *dai* onwards, the pathogen was abundant in all sections, with immature survival structures being identified in the cortex and parenchyma cells of the central cylinder at 7 *dai*, and mature chlamydospores separated from the somatic hyphae in cortex tissues and in the central cylinder at 14 *dai*. The oomycete in the cortex was found both in the apoplast and the symplast, whereas in the stele, the structures of the pathogen were found almost entirely inside the cells.

### Total Oomycete Structure Area (TSA)

**Figure 3:**
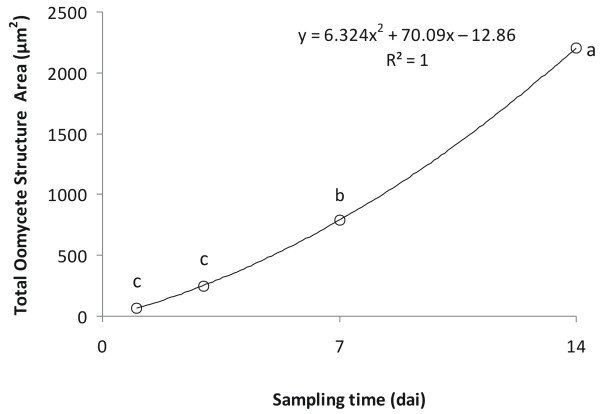
**Total oomycete structure area (TSA), with tendency curve.** Values with the same letter do not differ significantly (P < 0.001).

The analysis of variance depending on the sampling time showed highly significant (F = 199; P < 0.001) differences for this variable. The LDS analysis did not show significant differences between 1 and 3 *dai*, whereas there were significant (P < 0.001) differences at 7 and 14 *dai* (Figure 3).

The graphic representation of TSA showed a gradual positive increase in the slope. A quadratic equation (F = 102.959; P < 0.001) was shown to best fit the data (Figure 3).

### Area by type of structure

Significant differences were found for the following variables depending on the sampling time: *Intracellular Structure Area* (ISA) (F = 107; P < 0.001), *Extracellular Structure Area* (ESA) (F = 80; P < 0.001), and *Specific Structure Area* (SSA) (F = 27; P < 0.001).

**Figure 4:**
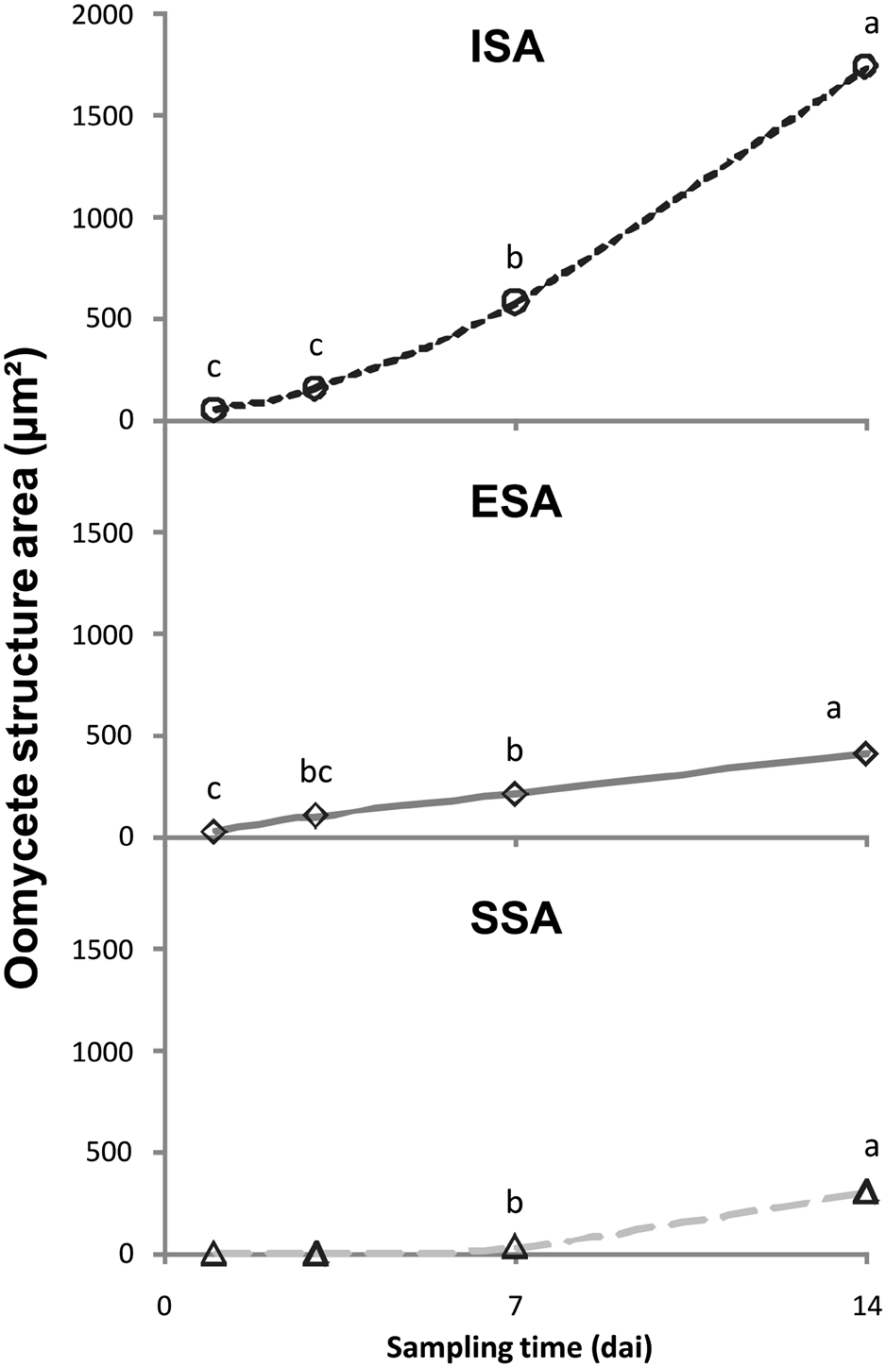
**Oomycete structure area, represented by different structure types (ISA, ESA and SSA).** Values with the same letter within a graph do not differ significantly (P < 0.001).

The ISA index for all sampling times was up to 80% of the TSA value, and its evolution throughout the sampling time was similar (Figure 4). The graphic representation of the ESA was linear, with limited slope, in which the test of class grouping only showed differences at 14 *dai* (Av = 183.44 μm^2^; P < 0.001). SSA does not show a value above zero until 7 *dai*, although after this time its increase was significant and reached similar values to ESA at the end of experience (14 *dai*).

## Discussion

The morphology of the hyphae matched the description reported for *P. cinnamomi* by the *European and Mediterranean Plant Protection Organization* (EPPO) [1]. The description of the haustoria-like structures agrees with published results by other authors [21,22]. Blaschke [22] observed the presence of this type of structure for *P. cinnamomi* in *Quercus robur* L. using *Scanning Electron Micrographs* (SEM). In a recent study, Brummer et al. [21] performed a *Transmission Electron Microscopy* (TEM) study and identified *Electronic Dense Material* (EdM) around the haustoria-like structures for *Phytophthora quercina* T. Jung in *Q. robur*. In both cases the visual description of these structures agrees with the results presented in the current study. However, in our case, further SEM and/or TEM studies are required to confirm whether these structures are haustoria or not. The specific structures and chlamydospores we describe are therefore similar to those described in other similar studies [1,15].

For all sampling times, extracellular and intracellular structures were detected, agreeing with the observations made by other authors [21-24]. Similar to the work conducted on *Corymbia calophylla* Lindl. (formerly known as *Eucalyptus calophylla* Lindl.) by Cahill et al. [24], the presence of the pathogen in the stele was detected at 24 h; however, contrary to the work conducted on *Q. ilex* and *Q. suber* L. by Pires et al. [23], in which they found a generalized invasion of the stele after this time, we only found scattered instances of hyphae in these tissues. However, details about the inoculum concentration or the growing conditions are not provided in the study of Pires et al. [23]. Cahill et al. [24] indicate the presence of immature survival structures at 72 h after inoculation, whereas in our study, these structures were not detected until 7 *dai*.

Clear pictures were obtained and the pathogen structures were sharp and well differentiated, so that both the sample preparation and the staining method used proved to be adequate for digital processing. The pathogen and host structures appeared to be well conserved for all of the sampling times. The differentiation between the pathogen and host structures were clear enough to allow for a selection of structures based on color gradient.

In the parenchymal tissues, the definition of the outline of the structures was more complex, due to the lower contrast in the captured images. The thickness of the transition of the outline varied by approximately 0.01 μm, whereas the smaller areas that were detected varied between 6 and 30 μm^2^, with the overall average area per structure being 45.8 μm^2^. Compared with the calculated areas, the potential variation between the micrographs and the computer generated structure images introduces an error of less than 0.01% of the overall average and was considered to be acceptable for this reason.

Regarding the measured variables (TSA, ISA, ESA and SSA), the appropriate adjustment of the normal distribution shows the absence of a bias. This observation indicated that the distribution of the variables is consistent with a random model, enabling us to conduct parametric tests, which is an important aspect for giving the methodology statistical robustness. Such tests revealed clear differences that explain, in a manner consistent with the visual observations, the evolution of infection and colonization in the root tissues. The exponential evolution of the TSA variable seems similar to the characteristic tendency of the growth of mycelial organisms [30,31]. This variable was the first to be obtained in our analysis and provides useful preliminary information about how the pathogen colonizes the root.

Consistent with the observations reported by other authors [22-24], we localized the oomycete invading the root by 24 h post-inoculation, spreading from the cortex through both the apoplast and the symplast by means of primary hyphae, in search of parenchymal tissues. From 3–7 *dai* on, colonization occurred more rapidly, as was previously observed in various *Quercus* species [21,22]. Once the oomycete recognizes these tissues, it increases its development, mainly growing through intracellular structures (hyphae and presumably, haustoria), which explains why the ESA index presents a lower growth rate in the advanced stages of colonization, relative to the increase in ISA. High levels of ESA alongside low levels of ISA show a pattern of tissue exploration by the pathogen. Low levels of ESA accompanied by a high ISA ratio show that the pathogen colonizes this tissue in order to obtain nourishment and to reproduce. Therefore, both indices, if they are examined together, can be useful for studying pathogen behavior in different plant tissues. The SSA index presents an important evolution between 7 and 14 *dai*, as it reaches, in half the time, the same level of presence in the root that it has in the extracellular structures. All of these data indicate that the pathogen during the advanced stages of colonization does not explore the tissue in a disorganized way but focuses on invading the parenchymal cells, to feed on them and to develop survival structures to complete its life cycle. The study of the SSA index may reveal the development status of the pathogen inside plant tissues. We might consider that the pathogen starts the development of chlamydospores and other specific structures when the colonization of tissue has extended and the oomycete expands into new areas of the roots.

## Conclusions

The determination of area parameters when studying the pathogen-host interaction in the root provides solid evidence that may help us quantify infection and colonization stages at the tissue level. This method provides a precise tool to evaluate hyphal colonisation and spore production in control and toxicity tests, using histological and microscopy techniques. In biological experiments, it is advisable to avoid techniques involving an added obstacle to analyze the results. The methodology presented here does not introduce higher levels of complexity, but provides results with an adequate statistical rigor.

The methodology presented in this study is readily accessible to staff with a basic knowledge of microscopy, histology and office automation, no complex mathematical models are required. Nevertheless, this approach generates results supported by rigorous statistical analyses and provides quantitative data to accompany the evolution that is observed in a qualitative manner during the development of infection and colonisation.

It must be noted that this method can be readily performed with the material that is available in a histology laboratory, as almost any image capturing equipment can be used, including a simple digital camera used together with a microscope.

The current method avoids using specific programs for histological analysis provided by firms that manufacture capturing devices, as these programs represent a considerable expense and often require specific training. Instead, the variables that are determined in this technique can be obtained using freely available image processing software, such as Wintopo Standard (Soft Soft Ltd, 2007, UK) or GIMP 2 (Free Software Foundation, Inc. 2008, USA), and assisted design programs based on CAD, such as FreeCAD (Juergen Riegel, Werner Mayer & Yorik van Harve, 2001–2011).

## Methods

### Plant material and growing conditions

The plant material was comprised of 6 month-old saplings of *Q. ilex* L. subsp*. ballota* (Desf.) Samp., grown from acorns gathered at “La Jarosa” farm (Córdoba, Spain. The Universal Truncated Mercator coordinates were 37,893-37,924 N, 4,945-4,901 W). The acorns were sterilized (5 minutes in a 1% NaClO solution), and germinated on perlite in a growth chamber (24°C, 70% relative humidity (RH), 12-h photoperiod). Seven days after germination, they were transferred to 32 cell seed trays containing 400 cm^3^ perlite and placed in a growth chamber (22°C, 60% RH, photoperiod 14 h of light/10 h in the dark), and watered twice weekly with Hoagland’s solution [32].

### Inoculation

Prior to inoculation the perlite was gently removed from the roots by washing in water, the roots of the samples were then submerged in a solution of liquid V8 medium that contained an A2-type isolate of *P. cinnamomi* (Pe-90), provided and certified by NBT laboratories (New Biotechnics, Sevilla, ISO 9001/17025). The pathogen was grown in Petri dishes containing 20 ml of V8 solution, in a growth chamber at 20°C in darkness. After 15 days the dishes were superficially washed and the mycelium in V8 medium was crushed in a mixer with distilled water (33 ml per dish). The final concentration of millet inoculum was adjusted counting chlamydospores as a reference and diluting it to 25 IU/μl.

After 10 minutes in the V8 medium, the samples were returned to the 32 cell seedling trays containing sterilized perlite, and were maintained in the growth chamber as described above. The control plants were not inoculated, but there roots were immersed in sterilized water.

### Sample processing

The inoculated plants were examined at 1, 3, 7, and 14 days after inoculation (*dai*) by taking samples from the absorbing roots (thin secondary rootlets of Ø ≤ 2 mm) that would exhibit the symptoms of infection described for root rot caused by *P. cinnamomi*[6,7,22,24,33]. At each sampling time, root samples from three inoculated plants and three control plants were harvested, avoiding root tips and lignified tissues. Twenty four plants were sampled in total (four sampling times, two treatments and three biological replicates). The sampled material was fixed in a paraformaldehyde-glutaraldehyde solution (Karnovsky solution), which is routinely used for ultrastructural microscopy studies [34,35], and was subsequently infused with synthetic resin using the Leica Historesin kit according to the protocols for dehydration and infusion with resin recommended by the manufacturer (Leica Microsystems GmbH, Wetzlar, Germany) (see Additional file 3).

The sampled root material was sectioned with a Leica RM 2245 Microtome (Leica Microsystems GmbH, Wetzlar, Germany) with carbon-tungsten knives Leica TC-65 (Leica Biosystems Nusloch GmbH; Geschäftsfürher, Germany). We obtained 2-μm thick longitudinal sections of the roots in a longitudinal disposition, which were then stained in a 0.1% toluidine blue-O solution in citrate buffer (pH 5) [35]. The sectioning thickness was chosen in order to obtain the maximum resolution and to minimize tissue destruction due to mechanical effects produced by the sectioning process. The samples were then mounted on a slide for optic microscopy with Entellan® synthetic resin for quick assembly (Merck KGaA, Darmstadt, Germany).

### Image capture and processing

From the pooled prepared samples, random subsamples were selected. The images were taken with lenses from the Plan Fluor series by Nikon Instruments Inc., through a Nikon DS-Fi1 digital optic device used with a Nikon Eclipse 50i optic microscope and connected to a PC through the Nikon DS-U2 control unity (Nikon Instruments Inc., Melville, NY, USA). The capture and storage of the images were performed with NIS-Elements F3.22.00 Build 710 computer software (Nikon Instruments Inc., 2008). A total of 160 images of 2560 x 1920 pixel were captured at 400X magnification.

**Figure 5:**
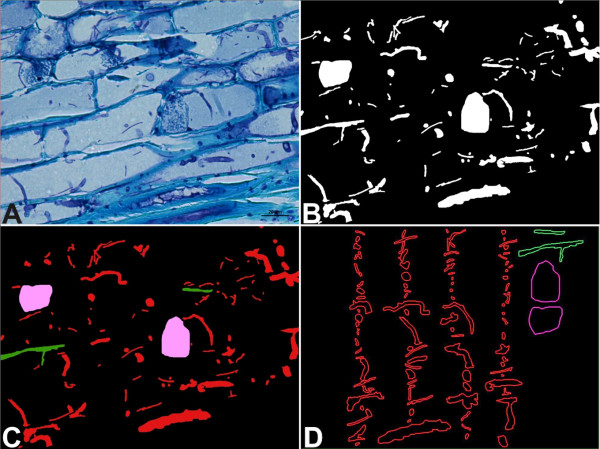
**Acquisition of measurable regions of pathogen colonization through image treatment. A**, Captured image from microscopy analysis. **B**, First representation map obtained from the subtractive masks, which represents the TSA. **C**, Color map after transformation (see Table 1 for Representative area color). **D**, Measurables structures in CAD files.

The images were treated with the CorelDRAW Graphics Suite 12 software package (Corel Corporation, 2003). The images were transformed into area maps of different colors using subtractive masks. On these maps, each type of structure was represented by a color (Figure 5).

The areas of the image belonging to the structures of the pathogen were differentiated from the rest of the plant tissue by the tones of the colors. We systematically scanned the images with a 22.58 x 22.58 pixel mesh (300 sectors), marking the pathogen structures present in each quadrant. We used a mask-editing tool included in the Corel Photo Paint software that identified the groups of pixels exhibiting color homogeneity within a determined range. This range was established in a 16-color interval for the standard 32-bit *Cyan, Magenta, Yellow & Key* (CMYK) color model. The outlines that were selected by this procedure were manually corrected when they exhibited a change in color or structure in contact with the cellular walls.

We thus obtained maps with five colors that were vectorized using a JPG image transformation tool with raster archives.

Finally, the area of each of the pathogen structures in the image was obtained by processing the maps that were vectorized with assisted design software (AutoCad 2009, AutoDesk Inc.). Thus, the following parameters shown in Table 1 were measured:

Total oomycete structure area (TSA): the area of all of the structures belonging to the pathogen and present in the sampled section.

Intracellular structure area (ISA): the area of the structures of the pathogen that were found within the cellular space.

Extracellular structure area (ESA): the area belonging to the structures of the pathogen that were not found inside the cells.

Specific structure area (SSA): the area of structures that are different from somatic hyphae and haustoria-like structures, which were not present before 7 *dai*. In this work, with *Phytophthora cinnamomi,* these include swollen hyphae, botryose hyphae, and chlamydospores. It does not include sporangiophores or oogonia because they were not found.

**Table 1:** Variables and Kolmogorov-Smirnov test results

**Variable**	**Representative area color**^**1**^	**Normality test**	
		**Kolmogorov -Smirnov**	**Sig**
**Total oomycete structures area (TSA)**	White	0.229	<0.001
**Intracellular structures area (ISA)**	Red	0.283	<0.001
**Extracellular structures area (ESA)**	Green	0.268	<0.001
**Specific structures area (SSA)**	Violet	0.424	<0.001

^1^See Figure 5B and C.

### Statistical processing

The normality of the studied variables was verified by the Kolmogorov-Smirnov test. Afterwards, an analysis of variance was conducted to determine the existence of significant differences and an LSD test of average grouping, establishing the significance level at 5%. The analysis was conducted with the SPSS 17 statistics package (SPSS Inc.).

## Competing interests

The authors declare that they have no competing interests.

## Authors’ contribution

FRG with RSC carried out the experiments under the supervision and guidance of RNC and APL, growing plants and inoculating roots, and performing the sampling procedure and histological preparations. FRG carried out the microscopy study, the image analysis of the sections, the statistical data analysis, and drafted the manuscript. APL participated in the development of the specific protocols and RNC with APL participated in the study design and helped to draft the manuscript. All authors read and approved the final manuscript.

## Supplementary Material

Additional file 1 **Figure S1. **Two series (A1-A5, B1-B5) of consecutive sections from vascular tissue of inoculated samples 14 ***dai.*** Observation of consecutive sections allows a three-dimensional reconstruction of the intracellular pathogen structures and confirms that the appearance of septate hyphae in a single section is an artefact. Bars present in figures A5 and B5 show 20 μm. Click here for file

Additional file 2 **Figure S2.** Schematic representation for the sectioning of several neighboring hyphae. When observed in a slide, a single section could be confused with a septated hypha. For that reason, several consecutive sections must be checked in order to get a three dimensional representation of the tissues. H1, Hyphae 1; H2, Hyphae 2; H3, Hyphae 3; H4, Hyphae 4. Click here for file

Additional file 3 Fixation, embedding and slide preparation protocols. Click here for file

## References

[B1] European and Mediterranean Plant Protection Organization Diagnostic protocols for regulated pests Bulletin OEPP/EPPO 2004 34 201 207

[B2] Hardam AR Pathogen profile: Phytophthora cinnamomi Mol Plant Pathol 2005 6 589 604 10.1111/j.1364-3703.2005.00308.x 20565682

[B3] Brasier CM Robredo F Ferraz JFP Evidence for Phytophthora cinnamomi involvement in Iberian oak decline Plant Pathology 1993 42 140 145 10.1111/j.1365-3059.1993.tb01482.x

[B4] Brasier CM Phytophthora cinnamomi and oak decline in Southern Europe. Environmental and constraints including climate change Annals of Forest Science 1996 53 347 358 10.1051/forest:19960217

[B5] Gallego FJ Perez de Algaba A Fernández-Escobar R Etiology of oak decline in Spain European Journal of Forest Pathology 1999 29 17 27 10.1046/j.1439-0329.1999.00128.x

[B6] Sánchez ME Caetano P Ferraz J Trapero A Phytophthora disease of Quercus ilex in South-Western Spain Forest Pathology 2002 32 5 18 10.1046/j.1439-0329.2002.00261.x

[B7] Sánchez ME Andicoberry S Trapero A Pathogenicity of three Phytophthora spp. causing late seedling rot of Quercus ilex ssp. Ballota Forest Pathology 2005 35 115 125 10.1111/j.1439-0329.2004.00392.x

[B8] Navarro-Cerrillo RM Terán AI Sánchez ME Acción Preventiva y Curativa del Fosfonato en el Control de Phytophthora cinnamomi Rands en Encina y Alcornoque Boletín de Sanidad Vegetal y Plagas 2006 32 685 694

[B9] Romero MA Sánchez JE Jiménez JJ Belbarhi L Trapero A Lefort L Sánchez ME New Pythium taxa causing root rot on Mediterranean Quercus Species in South-west Spain and Portugal Journal of Phytopatology 2007 155 289 295 10.1111/j.1439-0434.2007.01230.x

[B10] Ruizdela Torre J Flora Mayor 2006 Organismo Autónomo Parques Nacionales, Dirección General para la Biodiversidad, Madrid

[B11] Pérez A Cubera E Moreno G Solla A SEC F Evaluación de las Inyecciones de Fosfonato Potásico en un Foco de Seca de Extremadura Comunicación 5 Congreso Forestal Español 2009 Junta de Castilla y León, Valladolid 1 13

[B12] Maurel M Robin C Carpon G Desprez-Loustau ML Effects of root damage associated with Phytophthora cinnamomi on water relations, biomass accumulation, mineral nutrition and vulnerability to water deficit of five oak and chestnut species Forest Pathology 2001 31 353 369 10.1046/j.1439-0329.2001.00258.x

[B13] Robin C Carpon G Desprez-Lostau ML Root infection by Phytophthora cinnamomi in seedlings of three oak species Plant Pathology 2001 50 708 716 10.1046/j.1365-3059.2001.00643.x

[B14] Moralejo E García Muñoz JA Descals E Susceptibility of Iberian trees to Phytophthora ramorum and P. cinnamomi Plant pathology 2009 58 271 283 10.1111/j.1365-3059.2008.01956.x

[B15] Sánchez ME Sánchez JE Navarro R Fernández P Incidencia de la Podredumbre Radical causada por Phytophthora cinnamomi en Masas de Quercus en Andalucía Boletín de Sanidad Vegetal y Plagas 2003 29 87 108

[B16] Serrano MS Fernández P De Vita P Carbonero MD Trapero A Sánchez ME Lupinus luteus, a New Host of Phytophthora cinnamomi in Spanish Oak-Rangeland ecoystems Eur J Plant Pathol 2010 128 149 152 10.1007/s10658-010-9652-7

[B17] Solla A García L Pérez A Cordero A Cubera E Moreno G Evaluating potassium phosphonate injections for the control of Quercus ilex decline in SW Spain: implications of low soil contamination by Phytophthora cinnamomi and low soil water content on the effectiveness of treatments Phytoparasitica 2009 37 303 316 10.1007/s12600-009-0042-7

[B18] Navarro-Cerrillo RM Fernández P El Síndrome de la Seca del Encinar. Propuesta de Solución para el Valle de Los Pedroches 2000 Fundación Ricardo Delgado Vizcaíno, Pozoblanco

[B19] Navarro-Cerrilllo RM Fernández P Trapero A Caetano P Romero MA Sánchez ME Fernández A Sánchez I López G Los Procesos de Decaimiento de Encinas y Alcornoques. Sevilla 2004 Dirección General de Gestión del Medio Natural, Consejería de Medio Ambiente, Junta de Andalucía

[B20] Rodríguez-Molina MC Blanco-Santos EJ Palo Nuñez EJ Torres-Vila LM Torres Álvarez E Suárez-De-La-Cámara MA Seasonal and spatial mortality patterns of Holm oak seedlings in a reforested soil infected with Phytophthora cinnamomi Forest Pathology 2005 35 411 422 10.1111/j.1439-0329.2005.00423.x

[B21] Brummer M Arend M Fromm J Schlenzig A Obwald WF Ultrastructural changes and immunocytochemical localization of the elicitin quercinin inQuercus roburL. roots infected with Phytophthora quercina Physiological and Molecular Plant Pathology 2002 61 109 120 10.1016/S0885-5765(02)90419-4

[B22] Blaschke H Decline symptoms on roots of Quercus robur Euopean Journal of Forest Pathology (Forest Pathology) 1994 24 386 398

[B23] Pires N Maia I Moreira A Melo E Medeira C Vazquez-Pique J Early stages of infection of cork and holm oak trees by Phytophthora cinnamomi Suberwood: New Challenges for the Integration of Cork Oak Forests and Product 2008 Universidad de Huelva, Huelva 280 286

[B24] Cahill D Legge N Grant B Weste N Cellular and histological changes induced by Phytophthora cinnamomi in a group of plant species from fully susceptible to fully resistant Phytopathology 1989 79 417 424 10.1094/Phyto-79-417

[B25] Emeran A Sillero JC Fernández-Aparicio M Rubiales D Chemical control of faba bean rust (Uromyces viciae-fabae) Crop Prot 2011 30 907 912 10.1016/j.cropro.2011.02.004

[B26] Jagger LJ Newell C Berry ST MacCormack R Boyd LA Histopathology provides a phenotype by which to characterize stripe rust resistance genes in wheat Plant Pathology 2011 60 640 648 10.1111/j.1365-3059.2011.02436.x

[B27] Rubiales D Moral A Resistance of hordeum chilense against loose smuts of wheat and Barley (Ustilago tritici and U. nuda) and its expression in amphiploids with wheat Plant Breeding 2011 130 101 103 10.1111/j.1439-0523.2010.01818.x

[B28] Stark AK Gundersen HJG Gardi JE Pakkenberg B Hahn U The Saucor, a new stereological tool for analysing the spatial distribution of cells, exemplified by human neocortical neurons and glial cells Journal of Mycroscopy 2010 242 132 147 10.1111/j.1365-2818.2010.03447.x 21118392

[B29] Tschanz SA Burri PH Weibel ER A simple tool for stereological assesment of digital images: the STEPanizer J Microsc 2010 243 47 59 21375529 10.1111/j.1365-2818.2010.03481.x

[B30] Coelho AR Celli MG Sataque-Ono EY Wosiacki G Hoffman FL Pagnocca FC Yoko-Hirooka E Penicillium expansum versus Antagonist Yeast and Patulin Degradation in vitro Brazilian archives of Biology and Technology 2007 50 725 733 10.1590/S1516-89132007000400019

[B31] Taskin M Erdal S Genisel M Biomass and Exopolysaccharide production by Morchella esculenta in sumerged culture using the extract from waste loquat (Eriobotrya japonica L.) Kernels Journal of Food Processing and Preservation 2011 35 623 630 10.1111/j.1745-4549.2010.00510.x

[B32] Hoagland DR Arnon DI The water-culture method for growing plants without soil Circular 347 of California Agricultural Experiment Station 950 University of California, Berkeley (USA

[B33] Balci Y Balci S MacDonald WL Gottschalk KW Relative susceptibility to seven species of Phytophthora isolated from oak forest soils Forest Pathology 2008 38 394 409 10.1111/j.1439-0329.2008.00559.x

[B34] Pérez-de-Luque A Lozano MD Moreno MT Testillano PS Rubiales D Resistance to Broomrape (Orobranche crenata) in Faba Bean (Vicia faba): cell wall changes associated with prehaustorial defensive mechanisms Ann Appl Biol 2007 151 89 98 10.1111/j.1744-7348.2007.00164.x

[B35] Ruzin SĘ Plant Microtechnique and Microscopy 1999 Oxford University Press, New York

