# Supplementary figures and images for: A method to quantify infection and colonization of holm oak (Quercus ilex) roots by Phytophthora cinnamomi
Source: Plant Methods. 2012 Sep 13;8:39. doi: 10.1186/1746-4811-8-39 (PMC3495752; doi:10.1186/1746-4811-8-39)

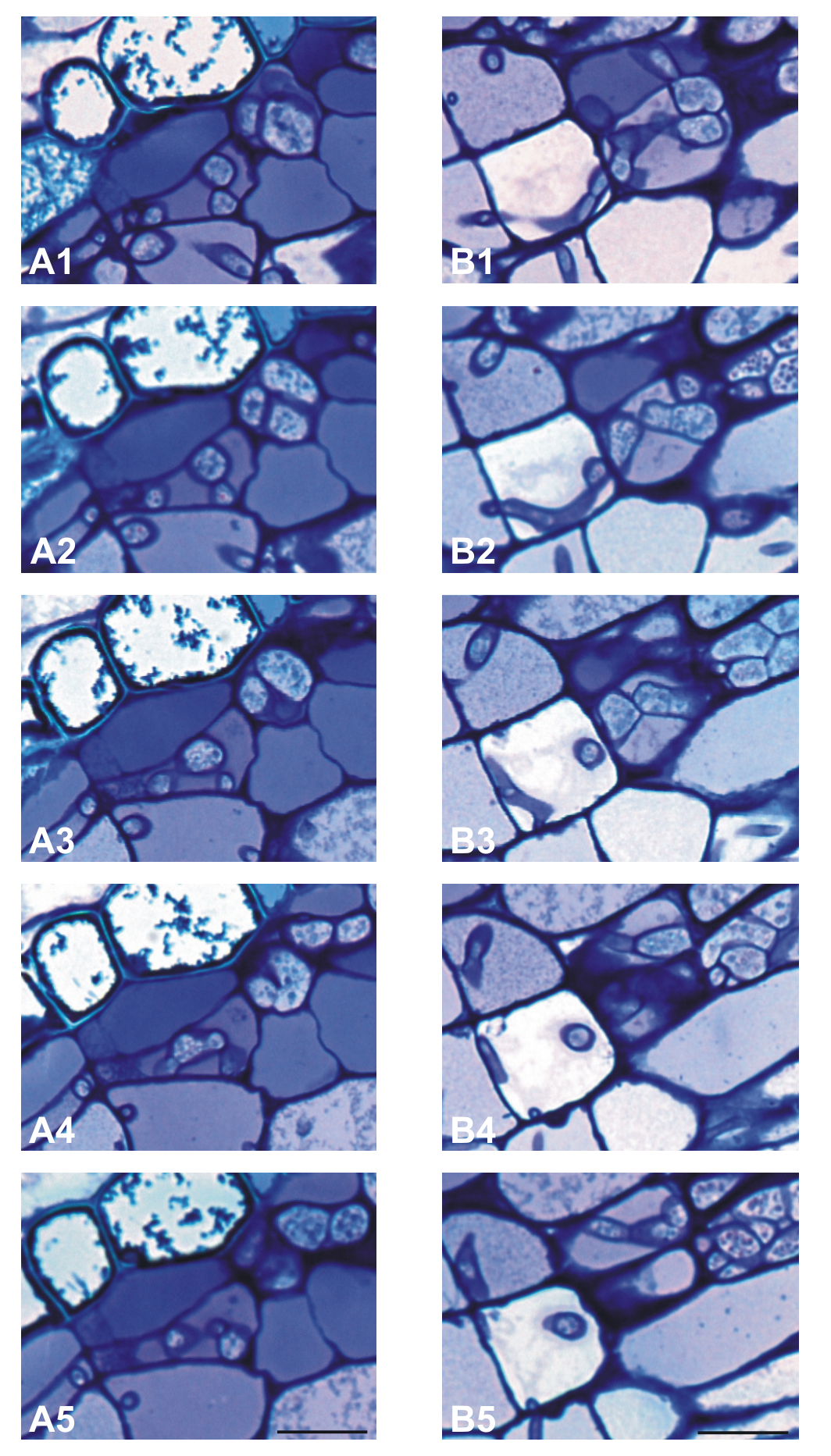

Supplement: Additional file 1 — Figure S1. Two series (A1-A5, B1-B5) of consecutive sections from vascular tissue of inoculated samples 14 dai. Observation of consecutive sections allows a three-dimensional reconstruction of the intracellular pathogen structures and confirms that the appearance of septate hyphae in a single section is an artefact. Bars present in figures A5 and B5 show 20 μm. [file 1746-4811-8-39-S1.png]

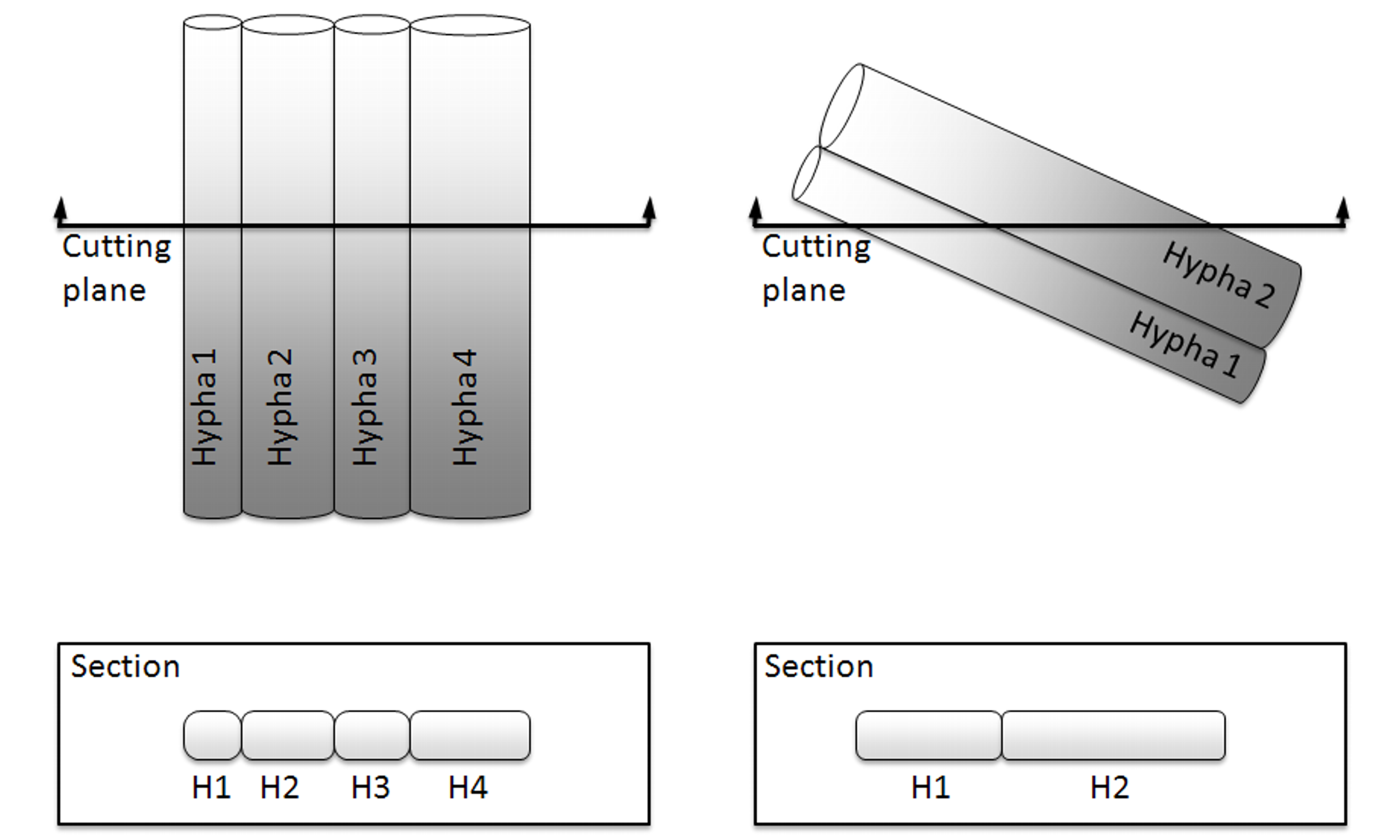

Supplement: Additional file 2 — Figure S2. Schematic representation for the sectioning of several neighboring hyphae. When observed in a slide, a single section could be confused with a septated hypha. For that reason, several consecutive sections must be checked in order to get a three dimensional representation of the tissues. H1, Hyphae 1; H2, Hyphae 2; H3, Hyphae 3; H4, Hyphae 4. [file 1746-4811-8-39-S2.png]
