# Supplementary material for: A method to quantify infection and colonization of holm oak (Quercus ilex) roots by Phytophthora cinnamomi
Source: Plant Methods. 2012 Sep 13;8:39. doi: 10.1186/1746-4811-8-39 (PMC3495752; doi:10.1186/1746-4811-8-39)
Supplement: Additional file 3 — Fixation, embedding and slide preparation protocols. [file 1746-4811-8-39-S3.doc]

**Karnovsky fixation**

(*Extracted from* Pérez-de-Luque *et al*., 2007)

1.-Materials

Karnovsky* Solution

Cacodylate buffer solution 0.025 M

Vacuum chamber

Fume hood

2 ml Microfuge tubes

1 ml Micrometric pipette

Hypodermic Syringe

250 ml Glass beaker

4ºC Refrigerator

2.-Karnovsky Solution *

Fixative for ultrastructure studies. Make the solutions under fume hood (Cacodylate is very toxic)

*2.1.-Materials*

Paraformaldehyde (CH_2_O)

Glutaraldehyde (C_5_H_8_O_2_)

Calcium chloride (CaCl_2_)

Sodium cacodylate (C_2_H_7_AsO_2_*Na)

Distilled water (or MiliQ water)

100 ml Volumetric Flask

100 ml Graduate Cylinder

Precision Scale

10 ml Volumetric glass pipette

*2.2.-Composition*

Paraformaldehyde 4%, Glutaraldehyde 5% in cacodylate buffer 0.025 M and 0.5 mg/ml Calcium chloride.

*2.3.-Preparation*

(From 25 ml)

- 12.25 ml paraformaldehyde8% (See below. Use fresh or freeze in aliquots for later use)
- Store at 4ºC.
- Add 0.0125 g Calcium chloride.
- Add 6.25 ml cacodylate buffer 0.1 M, pH 7.
- Add 5 ml glutaraldehyde 25 % (commercial buffer)
- Bring to 25 ml with distilled water.

*2.4.-Paraformaldehyde*

- Do not use commercial solutions. Make from powdered glutaraldehyde.
- Add 1g paraformaldehyde to 25 ml PBS buffer.
- Heat stirring, up to 60ºC (avoid overheating) until it becomes transparent. NaOH can be added to accelerate the process.
- Cool at 4ºC

If the solution is not going to be used immediately, then it must be frozen. Never use solutions prepared more than 24 hrs previously, unless they have been frozen.

3.-Plant samples fixation

Fix only fresh samples (fresh cut roots).

- Put some root portions of 0.5 cm length in 2 ml microfuge tubes, containing 1 ml Karnovsky solution.
- Apply 6 slight vacuum series of 1 min each, in a vacuum chamber, in order to facilitate infiltration of the fixative in the tissues. Store at 4ºC during 4 h.
- Washing: Replace Karnovsky solution by 0.025 M cacodylate buffer. After 15 min at room temperature, replace again the buffer solution. Wait another 20 min at room temperature, and replace one last time the buffer, storing at 4ºC in darkness.

**Resin embedding**

(*Extracted from user’s manual of Leica Historesin Embedding Kit ref. 7022 31731, and from* Pérez-de-Luque *et al*., 2007)

1.-Materials

Ethanol 96%

Distilled water

2 ml microfuge tubes

1 ml Micrometric pipette

Rotatory shaker

Infiltration solution*

Vacuum chamber

Binocular lens

Dissection tweezers

Microscope slides

Lancet or scalpel

Hardener Leica Historesin (Leica Mycrosistems Nußloch GmbH, Heidelberg, Germany)

Glass beaker

Pasteur Pipette

Synthetic mould blocks Leica Historesin Mold Tray L (Leica Mycrosistems Nußloch GmbH, Heidelberg, Germany)

Block Holders Leica Historesin Teflon Mold Trays (Leica Mycrosistems Nußloch GmbH, Heidelberg, Germany)

Needles

2.-Infiltration solution*

*Materials*

Basic resin Leica Historesin (Leica Mycrosistems Nußloch GmbH, Heidelberg, Germany) (2-hidroxyetil methacrylate, C_21_H_29_O_10_)

Basic resin activator Leica Historesin (Leica Mycrosistems Nußloch GmbH, Heidelberg, Germany) (Dibenzoylperoxyde, (C_6_H_5_CO)_2_O_2_)

100 ml ISO glass bottle

10 ml Volumetric glass pipette

Stirring magnets

Fume hood

Magnetic stirrer

Aluminium foil

*Solution*

Fill the glass bottle with 50 ml Basic resin and mix with one Basic resin activator packet, stirring in the fume hood. Wrap the bottle with aluminium foil and store at 4ºC for 12 h at least.

Do not use a solution older than 2 months.

3.-Sample dehydration and infiltration

Put the samples stored in 0.025M Cacodylate buffer in small tubes for dehydration through a solution series of 50, 80 and 95% ethanol in distilled water, remaining for 12 h in each solution.

Put the samples in 2 ml Microfuge tubes filled with 1 ml of 1:1 ethanol-infiltration solution. Keep stirring for 12 h.

After this, replace the solution in the tubes for 1 ml of pure infiltration solution. Apply a slight vacuum in vacuum chamber during 6 min. Store at 4ºC in darkness.

4.-Root sample resin embedding

*4.1.-Sample preparation*

Put the sample in a microscope slide with some drops of infiltration solution. Cut and clean samples with tweezers and lancet under the magnifier lens. Do not exceed 2-3 mm length.

4.2.*-Embedding*

Add 1 ml of hardener and 15 ml of infiltration solution in a flask. Shake gently with a pipette. The mixture will start to polymerize and harden in10 min. Before that, pour some mixture in the synthetic mould blocks and put the samples inside in the desired position with the help of needles. Put over the block holders and fill the moulds with mixture solution to the edge.

*4.3.-Block Extraction*

Complete resin polymerization takes about 2 h at room temperature. However, it is recommended to wait 48 h before extracting the block from the mould.

For extracting the blocks, make one incision in the block holder base, under the resin line, and pull from the block holder carefully. Before sectioning, let the blocks dry for a couple of days in order to eliminate the oily layer over the block surface.

**Staining and slide preparation**

(*Extract From* Pérez-de-Luque *et al*., 2007 *and from* Ruzin, 1999)

1.-Materials

Toluidine Blue-O (TBO) 0.1% in Citrate buffer (pH 5)*

Staining trays

Distilled water

Flat Tweezers

Filter paper

Synthetic slide assembly resin Entellan (butil 2-metilprop-2-enoate, C_13_H_22_O_4_) (Merck KGaA, Darmstadt, Germany)

Cover slip

2.-TBO 0.1 % *

2.1.-*Materials*

0.1 M citric acid solution

(Citric acid monohydrate C_6_H_8_O_7_*H_2_O [91.4% purity], 23 g/l)

0.1 M Sodium citratesolution

(Sodium citrate dihydrate C_6_H_5_Na_3_O_7_*2H_2_O [87.8% purity], 33.5 g/l)

TBO D.C. Panreac (Panreac Quimica S.A.U., Mollet del Valles, España)

[(C_15_H_16_ClN_3_S)_2_*ZnCl_2_]

Distilled water

100 ml Graduate Cylinder

3 ISO glass bottles autoclaved

Precision scale

Stirring magnets

Magnetic stirrer

2.2.-*0.1% TBO solution*

Mix in a glass bottle 41 ml 0.1 M citric acid solution with 59 ml of 0.1 M Sodium citrate solution and 100 ml distilled water (for 200 ml Citrate buffer).

Add 20 mg of TBO and stir using a magnetic stirrer.

3.-Staining procedure

Introduce the dry slides containing the sample sections in one staining tray with 0.1% TBO for 5 min. Then change the slides into a staining tray containing distilled water. Wash the slide shaking slightly and put into another staining tray with clean distilled water for 5 min. Repeat the process. After that, let the slides to dry overnight on a filter paper at room temperature.

4.-Slide assembly

Using a glass rod, put 3 to 4 drops of synthetic resin over the slides. Then, put the cover slip avoiding bubbles formation. Dry overnight at room temperature.

References:

- Pérez-de-Luque A, Lozano MD, Moreno MT,Testillano PS,Rubiales D:**Resistance to Broomrape (*Orobranchecrenata*) in Faba Bean (*Viciafaba*): Cell Wall Changes Associated with Prehaustorial Defensive Mechanisms.** *Annals of Applied Biology* 2007,**151**:89-98.
- Ruzin, S.Ę: *Plant Microtechnique and Microscopy.*New York: Oxford University Press; 1999.
